# Mussel-Inspired Fabrication of PDA@PAN Electrospun Nanofibrous Membrane for Oil-in-Water Emulsion Separation

**DOI:** 10.3390/nano11123434

**Published:** 2021-12-17

**Authors:** Haodong Zhao, Yali He, Zhihua Wang, Yanbao Zhao, Lei Sun

**Affiliations:** 1 Engineering Research Center for Nanomaterials, Henan University, Kaifeng 475004, China; zhd880880@163.com (H.Z.); hnsunlei@163.com (Y.H.); zhaoyb902@henu.edu.cn (Y.Z.); 2 Henan Engineering Research Center of Industrial Circulating Water Treatment, College of Chemistry and Chemical Engineering, Henan University, Kaifeng 475004, China

**Keywords:** polyacrylonitrile, electrospinning, nanofibrous membrane, emulsion separation

## Abstract

Emulsified oily wastewater threatens human health seriously, and traditional technologies are unable to separate emulsion containing small sized oil droplets. Currently, oil–water emulsions are usually separated by special wettability membranes, and researchers are devoted to developing membranes with excellent antifouling performance and high permeability. Herein, a novel, simple and low-cost method has been proposed for the separation of emulsion containing surfactants. Polyacrylonitrile (PAN) nanofibers were prepared via electrospinning and then coated by polydopamine (PDA) by using self-polymerization reactions in aqueous solutions. The morphology, structure and oil-in-water emulsion separation properties of the as-prepared PDA@PAN nanofibrous membrane were tested. The results show that PDA@PAN nanofibrous membrane has superhydrophilicity and almost no adhesion to crude oil in water, which exhibits excellent oil–water separation ability. The permeability and separation efficiency of n-hexane/water emulsion are up to 1570 Lm^−2^ h^−1^ bar^−1^ and 96.1%, respectively. Furthermore, after 10 cycles of separation, the permeability and separation efficiency values do not decrease significantly, indicating its good recycling performance. This research develops a new method for preparing oil–water separation membrane, which can be used for efficient oil-in-water emulsion separation.

## 1. Introduction

With an increase in oil spills and sewage discharge, water pollution has become an increasingly serious environmental problem. Therefore, water separation technology has received more and more attention in the past decades [1,2,3]. The type of oil in water is divided into three types according to their physical state: free oil (oil droplets with the size bigger than 150 μm), dispersed oil (oil droplets with the size between 20 and 150 μm) and emulsified oil (oil droplets with the size smaller than 20 μm), of which emulsified oil processing is the most difficult [4,5,6]. Currently, various conventional physical and chemical methods have been applied for removing oil from oily wastewater, such as gravity-based technologies [7], coagulations and floating [8,9], oil skimmers [10], adsorbent materials [11] and filtration [12], etc. However, these traditional separation methods have several disadvantages, such as high energy cost, complicated operation process and secondary pollution. In addition, most of them are ineffective in treating emulsified oil [13,14]. Furthermore, well-dispersed oil-in-water emulsion is usually destabilized by adding demulsifying agent, which is expensive and environmentally unfriendly [15]. In general, it is difficult to separate emulsified oil–water mixtures by conventional methods and materials because the size of the oil droplets in the oil–water emulsion is usually less than 20 μm, and the oil droplets are stably and uniformly dispersed in water [16,17]. These emulsion droplets range in sizes from a few hundred to thousand nanometers, and their surface tensions are lower than those of surfactant-free emulsified oil [18]. Therefore, separating emulsified oil droplets from surfactant-containing water remains challenging. Efforts should be made to design advanced separation materials with high efficiency and low price to separate emulsified oil, which is the focus and difficulty of future research.

Electrospun fiber membrane has gained attention in separation applications over the past ten years. The nanofibers made by electrospinning have many advantages, such as higher porosity (usually about 80%), large surface area (up to 40 m^2^/g depending on the fiber diameter) [15], continuously interconnected pores, adjustable shape, tunable wettability and multifunctional components [19,20]. In addition, the electrospun fibers’ diameter are controllable from micrometers to a few nanometers, which is suitable for effective separation for the emulsified oil–water mixture [21]. The most commonly used membrane materials, such as polyvinylidene fluoride (PVDF) [22], polysulfone (PSF) [23], polytetrafluoroethylene (PTFE) [24] and polypropylene (PP) [25] are hydrophobic. These membranes are susceptible to oil contamination during the separation process, which greatly limits their suitability [26]. In fact, the development of super hydrophilic, super oleophobic underwater surfaces provides a viable method for oil-in-water emulsion separation [27]. Hydrophilicity allows water to quickly penetrate membranes, and underwater oleophobicity hinders oil droplets from passing through the membrane, thereby successfully separating oil-in-water emulsions [28]. For example, Kong et al., prepared a hygro-responsive super oleophobic/super hydrophilic coating by liquid deposition of TiO_2_ with perfluorooctanoic acid. The hygro-responsive coating can separate different types of oil–water mixtures with a separation efficiency of more than 99% [29]. Yang et al. prepared a novel graphene oxide linked cotton fiber (GOCCF) membrane. GOCCF membrane has high separation efficiency for all kinds of oil–water separation driven by gravity, and its separation efficiency remains above 99.8% [30]. However, these materials are not as ideal as electrostatic spinning fiber membranes in terms of porosity and specific surface area.

Polyacrylonitrile (PAN), a polymer with acrylonitrile as the repeating unit, has been widely used in ultrafiltration, nanofiltration, and reverse osmosis membranes due to their high chemical resistance, thermal stability and excellent wettability with water [31,32]. polydopamine (PDA) has often been used as a modifier for improving the hydrophilicity and reactivity of substrates [33]. The invention of PDA coatings is inspired by the bonding properties of marine mussels [34,35,36]. It is well known that the strong adhesion of PDA coatings to various substrates can be attributed to interactions between covalent and non-covalent catechol and amine structures in autoxidation polymerization [37,38].

**Figure 1:**
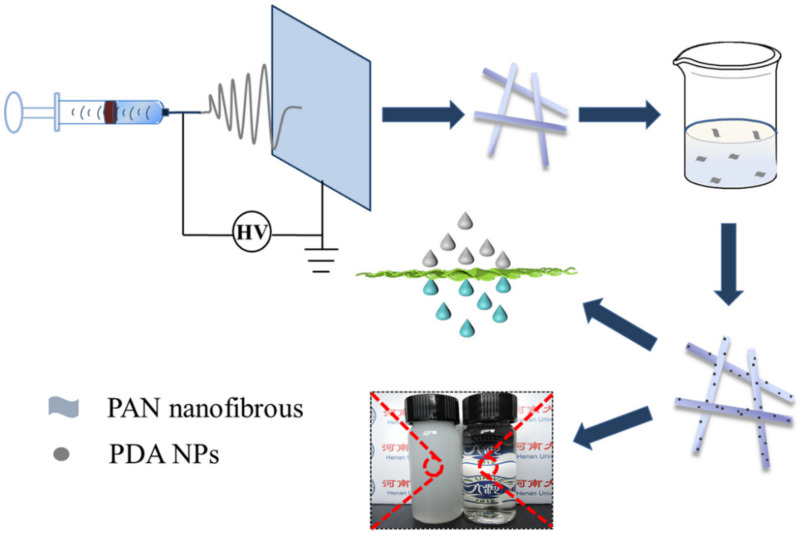
Schematic diagram of preparation process and oil-in-water emulsion separation performance of PDA@PAN electrospinning nanofibrous membrane.

In this study, we prepared a novel super hydrophilic, super-oleophobic underwater oil/water separation membrane. Firstly, a PAN nanofibrous membrane was prepared by electrospinning and then the self-polymerization time of dopamine was controlled to form PDA nanoclusters, which deposited on PAN nanofibers. The final product is named the PDA@PAN electrospun membrane. The as-prepared PDA@PAN electrospun membrane exhibits hydrophilicity in air and lower oil adhesion underwater. In addition, it has excellent separation efficiency for emulsion composed of different organic phase such as n-hexane/water, n-dodecane/water and n-hexadecane/water. The schematic diagram of preparation process and oil-in-water emulsion separation performance of PDA@PAN electrospinning nanofibrous membrane is shown in Figure 1.

## 2. Materials and Methods

### 2.1. Reagents

PAN (MW = 150,000) was obtained from Shanghai Macklin Biochemical Technology Co., Ltd. (Shanghai, China). Dopamine hydrochloride (DA), sodium dodecyl sulfate (SDS), aminomethane hydrochloride (Tris-HCl), oil red O (C_26_H_24_N_4O_) and n-hexadecane were purchased from Aladdin Biological Technology Co., Ltd. (Shanghai, China). N, N-dimethylformamide (DMF) was supplied by Kemiou Chemical Reagent Co., Ltd. (Tianjin, China). Chloroform was provided by Luoyang Haohua Chemical Reagent Co., Ltd. (Luoyang, China). N-hexane was purchased from Tianjin Fuyu Fine Chemical Co., Ltd. (Tianjin, China). N-dodecane was obtained from Shanghai Lingjin Fine Chemical Co., Ltd. (Shanghai, China). With the exception of specially labeled reagents, these reagents are of analytical grade (AR) and were used without further purification.

### 2.2. Instruments and Characterization

The electrospun device was set up by ourselves, which consisted of a high voltage power supply (DE-200) and a voltage-stabilized source (POWER-01) purchased from Dingtong Science & Technology Development Co., Ltd. (Dalian, China); a syringe pump (LSP02-1B) purchased from Longer Precision Pump Co., Ltd. (Baoding, China); and an electrically grounded collector. Scanning electronic microscopy (SEM) images were obtained by using a scanning electron microscope (FEI Nova NanoSEM 450, Thermo Fisher Scientific, Waltham, MA, USA) at an acceleration voltage of 30 kV, a working distance of 5 mm and a spot size of 3.0 nm. An energy-dispersive X-ray spectroscope (EDX, X-MaxN, OXFORD Instruments, Oxford UK) was connected with SEM for elemental analysis. An X-ray photoelectron spectrometer (XPS, ESCALAB 250XI, Thermo Fisher Scientific, Waltham, MA, USA) was used to confirm the surface chemical composition of PDA@PAN electrospun nanofibrous membrane. Fourier transform infrared spectroscopy (FTIR) was measured with a Fourier transform infrared spectrometer (AVATAR360, Nicolet Instrument Corporation, Richardson, TX, USA) in a wavenumber range of 400–4000 cm^−1^. The optical microscopy images of the oily emulsion samples were observed by an optical microscope (XSP-15CE, Changfang Optical Instrument Co., LTD., Shanghai, China). Contact angle tests of water and other liquids were measured on a DSA-100S optical contact-angle meter (Kruss, Hamburg, Germany) at room temperature. The average contact angle value was determined by measuring the same sample at five different positions. All optical photos were taken with a digital camera (P600, NIKON Corporation, Tokyo, Japan). The average total organic carbon (TOC) value in the feed solution and the corresponding filtrate were measured by using a total organic carbon analyzer (TOC-L, Shimadzu Corporation, Kyoto, Japan).

### 2.3. Preparation of PAN Nanofibrous Membrane

The PAN electrospun nanofibrous membrane was prepared as follows [39]: Before the electrospinning process, 0.5 g PAN was dissolved in 4.5 g DMF solution and kept stirring at 25 °C for 24 h to obtain a light-yellow transparent solution. Then, the as-prepared 10 wt% PAN electrospun solution was loaded into a 10 mL plastic syringe posessing a 17-gauge stainless steel needle with a 90° blunt end. The feeding rate of the spinning solution was 0.7 mL/h, and the distance from the spinneret to the collector was 15 cm. High pressure, measuring 15 kV, was applied to the tip of the needle, resulting in a continuous jet. The temperature was maintained at 35 ± 5 °C, and relative humidity was controlled at 40 ± 5%. The resulting PAN electrospun nanofibers were collected on electrically grounded aluminum foil. Finally, the as-prepared PAN nanofibrous membrane was dried in a vacuum oven at 40 °C for 4 h in order to completely dry it.

### 2.4. Fabrication of PDA@PAN Nanofibrous Membrane

Firstly, the as-prepared PAN nanofibrous membrane was cut into pieces at a size of 3 m× 3 m. Then, it was immersed into 20 mL of 10 mmol/L Tris-HCl solution (pH = 8.5). Secondly, 40 mg of DA was added to the solution, and the mixture was placed in a 135 r/min shaker at a temperature of 25 °C for 7 h. As expected, DA self-polymerized into PDA nanoparticles and became attached to the surface of PAN nanofibrous membranes. Finally, the as-prepared PDA@PAN membrane was washed several times with distilled water to remove free PDA nanoparticles and then dried in an oven at 45 °C for 5 h.

### 2.5. Oil-In-Water Emulsion Separation Test

In order to verify the separation capacity of the as-fabricated PDA@PAN membrane for oil-in-water emulsion, three different oil-in-water emulsions (i.e., n-hexane, n-dodecane and n-hexadecane) were prepared previously, and SDS was used as an emulsifier for them. As a typical procedure, 3.0 mg SDS was dissolved in 300 mL distilled water. Then, 10 mL n-hexane was injected into the solution immediately. The mixed solution was dispersed by ultrasonic for 1 h at ambient temperature to obtain a homogeneous milky emulsion. The other two surfactant-stabilized oil-in-water emulsions were formulated in the same oil–water ratio and SDS concentration.

The oil-in-water emulsion separation process was conducted as follows: First of all, a set of separation devices was set up. The separation equipment consisted of the upper feed pipe, the middle connecting pipe and the bottom conical flask collector. At the same time, the vacuum pump was connected to the suction port of the intermediate tube in order to provide the required vacuum conditions. In the oil-in-water emulsion separation procedure, a piece of the as-prepared PDA@PAN nanofibrous membrane was cut and placed on a quartz sand filter, which is fixed into the connecting pipe with an inner diameter of 25 mm and equipped with a suction mouth. Before separation, the PDA@PAN nanofibrous membrane was soaked by distilled water so that it closely covered the filter tablet, and the collector container was vacuumed to 0.08 MPa. After that, 30 mL oil-in-water emulsion was poured into the upper feed tube. Immediately, transparent filtrate flowed into the bottom conical flask with steady flow due to the separation effect of the as-prepared PDA@PAN nanofibrous membrane.

In order to verify the recyclability of the as-prepared PDA@PAN nanofiber membrane, a cyclic test was carried out on the separation of n-hexadecane aqueous emulsion. After each separation, the membrane was washed with distilled water in order to remove adhesive oil droplets and then dried in an oven at 60 °C for 20 min, followed by utilization in the next separation cycle. Separation was repeated for 10 cycles.

Efficiency is one of the most important abilities for membrane separation, which can be evaluated by using Equation (1) [40]:

(1) R=1−CfCo×100%

where R denotes oil rejection, C_o_ (mg/L) denotes oil value in the original oil-in-water emulsion and C_f_ (mg/L) is the residual oil value in the filtrate after separation. C_o_ and C_f_ are TOC values of feed and filtrate solutions.

The water permeability is another significant capacity for separation membrane, which is calculated using Equation (2) [41]:

(2) J=VA∆tP

where J (Lm^−2^ h^−1^ bar^−1^) denotes the water permeability of the membrane, V (L) is the volume of filtrate, A (m^2^) is the effective filtration area of membrane, Δ_t_ (h) is the testing time and P (bar) is the operating pressure.

Before and after separation, feed and filtrate solutions were observed by an optical microscope in order to obtain visualized images of emulsified small droplets so as to show the separation ability of the membranes in a visible manner.

## 3. Results and Discussion

### 3.1. Morphology and Structure of PDA@PAN Nanofiber Membrane

**Figure 2:**
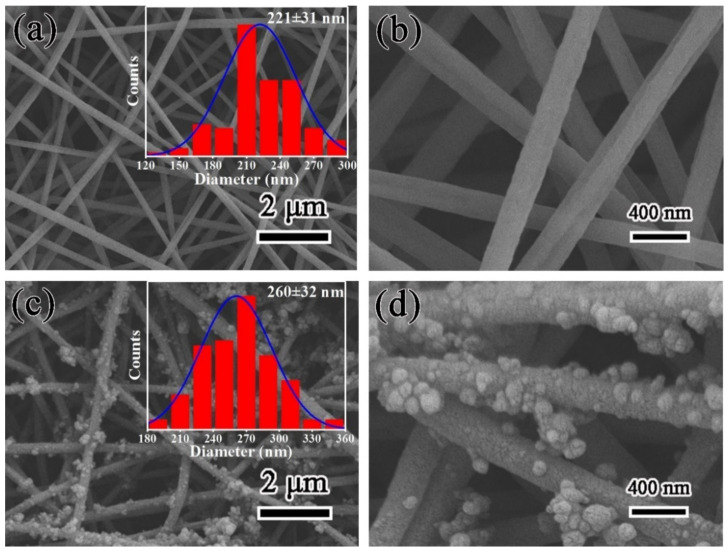
SEM images of PAN (**a**,**b**) and PDA@PAN nanofirous membrane (**c**,**d**); the insets in (**a**,**c**) are histograms of nanofibers diameter distribution.

The surface morphology of the as-prepared PAN and PDA@PAN nanofibrous membrane were analyzed by scanning electron microscopy. Figure 2 shows the SEM images of PAN (a,b) and PDA@PAN nanofibrous membrane (c,d), the insets are histograms of nanofiber diameter distribution. As shown in Figure 2a,b, PAN nanofibers have a uniform and smooth surface. The average diameter of PAN nanofibers is determined to be 221 ± 31 nm by using particle size statistics software. The long, continual and intertwined PAN nanofibers are stacked randomly to form a three-dimensional porous membrane with high porosity, which is essential for the formation of water channels and efficient separation of emulsions. However, it is obvious observed from Figure 2c,d that after immersion in polydopamine solution and drying, the surface of PDA@PAN nanofibers is no longer smooth, and particles are attached. These attached particles are uneven and sparsely distributed on the fibers surface. However, due to the dense and disordered heap of fibers, the modified PDA nanoparticles should have a uniform and dense load in the overall structure of the fibrous membrane, which is beneficial for improving the surface super hydrophilicity of the sample. Moreover, from the diameter distribution histogram inserted in Figure 2c, it is observed that the diameter of PDA@PAN nanofibers is in the range of 260 ± 32 nm. Compared to unmodified PAN nanofibers, the fiber diameter is significantly increased, which further indicates PDA coated on the PAN nanofibers surface. It is worth mentioning that PDA has a similar molecular structure to 3,4-dihydroxyphenylalanine (DOPA), which is the main component of mussel mucin excreted by Marine mussels. Due to of crosslinking between catechol and amino groups in its molecules and strong chelation with the substrate, PDA shows strong adhesion. Thus, it can tightly adhere to PAN substrates and is favorable for maintaining its hydrophilicity.

**Figure 3:**
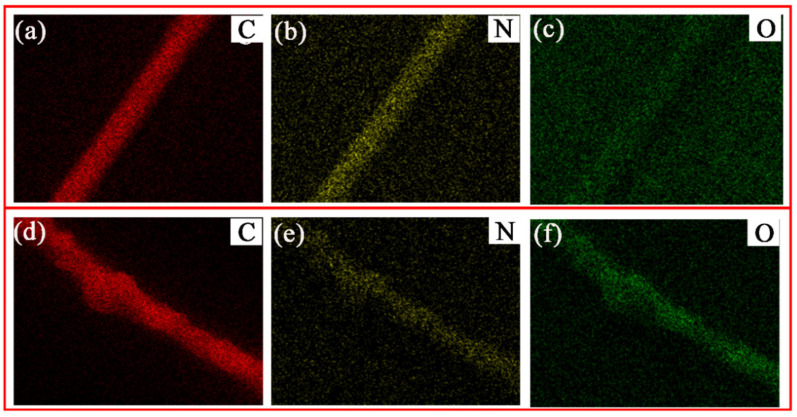
EDX maps of C, N and O elements in PAN (**a**–**c**) and PDA@ PAN (**d**–**f**) nanofibers.

In order to further determine the chemical composition of the PAN and PDA@PAN electrospun nanofibrous membrane, we studied the element distribution in the fiber with EDX, and a silicon wafer was used as the substrate in the test. Figure 3 shows the C, N and O elements mapping in PAN (a–c) and PDA@PAN(d–f) nanofibers. It can be observed from Figure 3a–c that the presence of C, N and O was detected on unmodified PAN electrospun nanofibers with EDX. Among them, C and N signals are strong and distributed along the outline of a single nanofiber, which comes from the carbon chain and nitrile group in PAN; the signal of O is weak, and although the molecular structure of acrylonitrile does not contain O atoms, the PAN reagents used are usually binary or terpolymers. Trace O comes from the oxygen-containing groups of other polymerized monomers. As shown in Figure 3d–f, the EDX distribution of PDA@PAN nanofibers still shows the presence of three elements C, N and O, but compared with unmodified fibers, the distribution of C and N on the fiber surface shows a definite heave along the fiber direction, which can be attributed to the strong adhesion of modifier PDA nanoparticles. Moreover, the signal intensity of O increased, since the introduced PDA contains O atoms (derived from the o-diphenol group on the benzene ring). EDX analysis results strongly verify that PDA was modified on the surface of PAN fibers.

**Figure 4:**
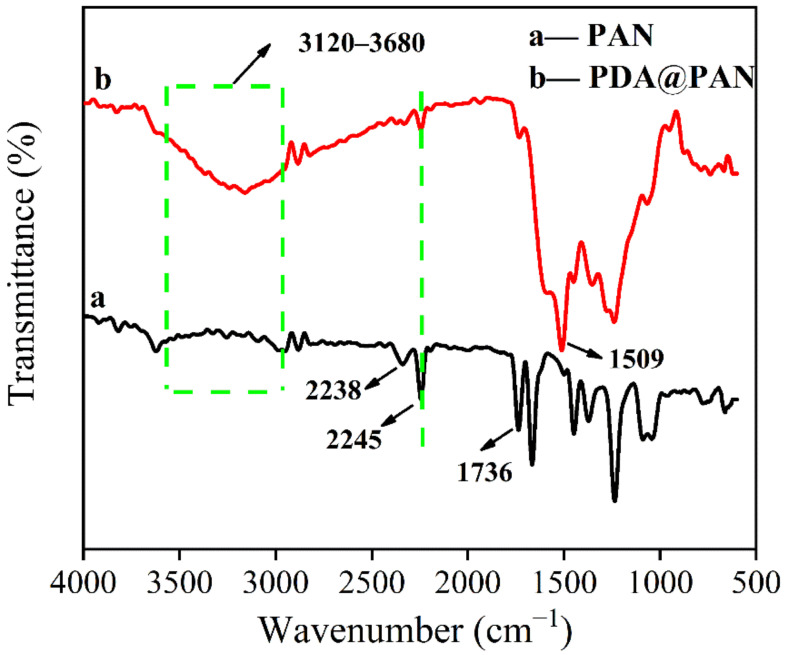
FTIR spectra of PAN (a) and PDA@PAN (b) nanofibrous membrane.

Furthermore, FTIR characterization was conducted to confirm whether PDA is modified on the surface of PAN nanofibers. Figure 4 is the FTIR spectra of PAN (a) and PDA@PAN (b) nanofibrous membrane. It should be specifically pointed out that an attenuated total reflection (ATR) mode was applied in the test. For PAN samples, the absorption peak at 2245 cm^−1^ is attributed to the tensile vibration of the nitrile carbon–nitrogen triple bond in PAN [42]. While the absorption peak at 1736 cm^−1^ may be ascribed to the stretching vibration of the ester carbonyl group in polymethyl methacrylate (PMMA), which is a minor component in the copolymer [43]. Compared to the FTIR spectra of PDA@PAN and unmodified PAN nanofibrous membranes, it was observed that the C≡N absorption peak of the PAN nitrile group was retained at 2245 cm^−1^, but new absorption bands appear at 3120–3680 cm^−1^ and 1509 cm^−1^, which are attributed to the stretching vibrations of the phenolic hydroxyl group and the bending vibrations of the N-H bond in the PDA [44,45]. This analysis further confirmed that the surface of PAN nanofibers was coated by PDA.

**Figure 5:**
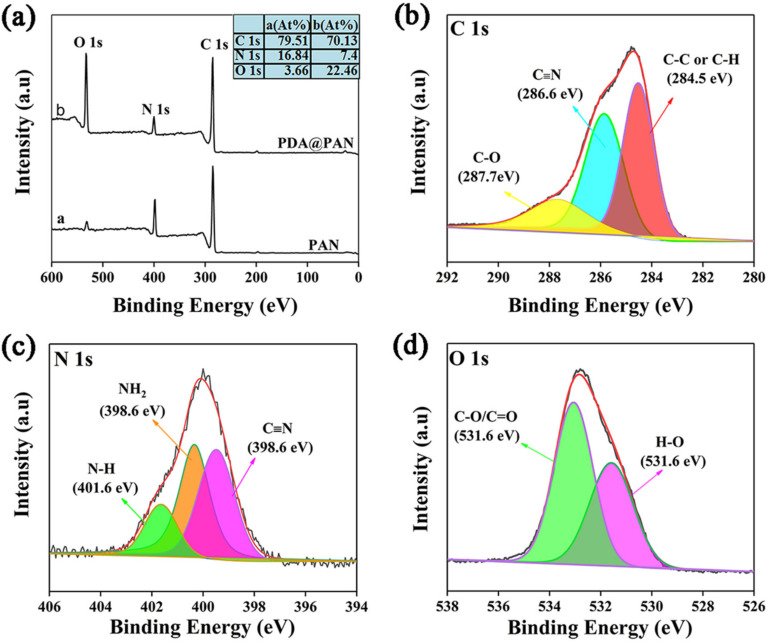
XPS full spectra of PAN and PDA@PAN nanofibrous membrane (**a**) and deconvoluted XPS spectra of C 1s (**b**), N 1s (**c**) and O 1s (**d**) elements; the inset table in (**a**) shows relative atomic percentage.

The chemical environment of the surface atoms of the fibrous membrane was further analyzed by XPS to determine the successful coating of PDA. Figure 5a shows the XPS full spectra of PAN and PDA@PAN nanofiber membranes, and the inset is a table of relative atomic percentage. It can be observed from Figure 5a that the two membrane samples have XPS peaks of C 1s, N 1s and O 1s emerging at electron binding energies of 284.9, 399.1 and 531.5 eV, respectively [46]. From the inserted relative atomic percentage table, it can be found that content of O 1s increased from 3.66% to 22.46% after PDA modification, which is consistent with previous EDX and FTIR analysis results. At the same time, it is observed that the ratio of O/C atomic content is also significantly increased compared PDA@PAN to that of the PAN nanofirous membrane, which is also a result of the modification of PAN nanofibers by PDA.

Figure 5b–d show deconvoluted XPS spectra of C, N and O in PDA@PAN nanofibrous membrane. As shown in Figure 5b, the XPS spectra of C 1s were resolved into three peaks at 287.7, 286.6 and 284.5 eV, which belong to different groups where the C atom is present, namely C-O, C≡N, C-C or C-H. Similarly, the three peaks of N 1s at 398.0, 399.8 and 400.9 eV are, respectively, attributed to C≡N, NH_2_ and N-H groups in the PDA@PAN membrane (as shown in Figure 5c) [46]. Finally, from Figure 5d, it is observed that XPS spectra of O 1s are divided into two peaks, appearing at 531.6 and 533.0 eV, and correspond to oxygen-containing functional groups of H-O and C-O/C=O [47]. They are derived from the phenol group in PDA and the ester carboxyl group in the copolymer PMMA [48].

### 3.2. Hydrophilicity of PDA@PAN Nanofibrous Membrane in Air

**Figure 6:**
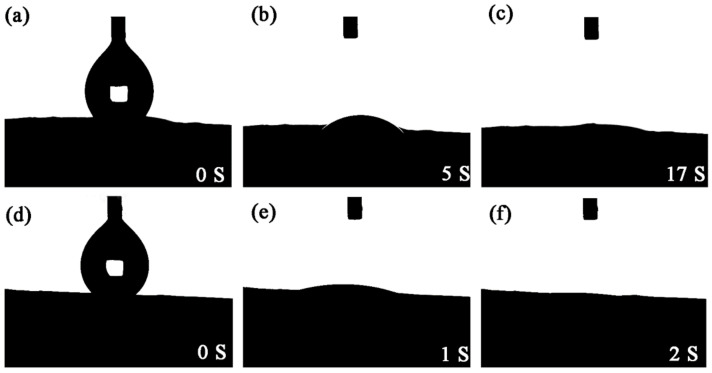
Photos of dynamic measurements of water spreading for PAN (**a**–**c**) and PDA@PAN (**d**–**f**) electrospun nanofibrous membrane.

The high-speed camera system of the hydrophobic angle tester was used to record the dynamic hydrophilic behavior of the nanofibrous membrane in the air to study the surface wettability of the PDA@PAN membrane in the air. Figure 6 shows the photos of the dynamic measurement of water spreading for PAN (a–c) and PDA@PAN (d–f) electrospun nanofibrous membrane. As shown in Figure 6a–c, when 4 μL of water droplets was slowly dropped onto the surface of PAN nanofibrous membrane in the air, the water droplets completely infiltrated and spread within 17 s, and the contact angle reaches 0°, which indicates that the PAN nanofibrous membrane has a certain degree of hydrophilicity [49]. However, it can be observed from Figure 6d–f that the contact angle of water droplets on the PDA@PAN nanofibrous membrane can quickly become 0° within 2 s, indicating that the surface is extremely hydrophilic. By comparing the results of dynamic diffusion of water droplets, it can be observed that the hydrophilicity of PAN nanofiber membrane improved obviously after PDA modification, thus obtaining superhydrophilicity [50].

### 3.3. Underwater Oleophobicity of PDA@PAN Nanofiber Membrane

**Figure 7:**
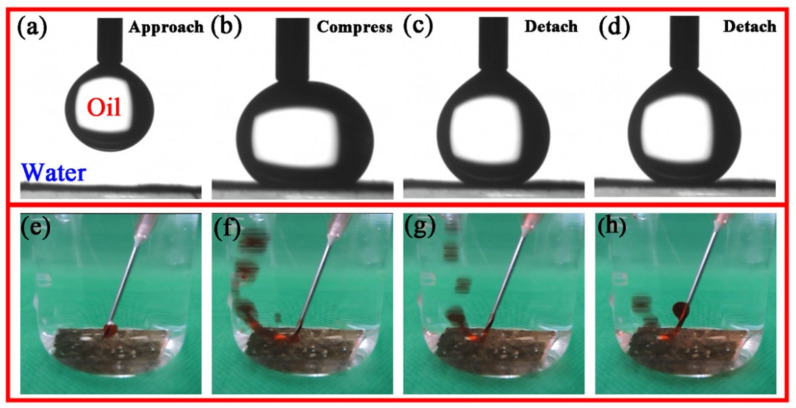
Photos recording underwater measurement of PDA@PAN nanofirous membrane droplet resistance (**a**–**d**) and oil-resistance performance (**e**–**h**) in motion.

In order to separate the oil-in-water emulsion successfully, the as-prepared filter membrane material should have excellent underwater oleophobicity in addition to hydrophilicity in the air. For this purpose, underwater oil repellent and anti-oil adhesion experiments were designed. Figure 7a–d show dynamic pictures of the underwater resistance of PDA@PAN nanofibrous membranes to oil droplets. The specific experimental process and phenomena are as follows: Firstly, PDA@PAN nanofibrous membrane was fixed to the substrate and placed at the bottom of a quartz beaker filled with distilled water. Chloroform was selected as representative oil and added into a micro syringe. After the needle was inserted into the water, an oil drop was extruded and suspended on the film (Figure 7a). Subsequently, the oil droplet moved downwards slowly and was squeezed until it was deformed by touching the surface of the film (Figure 7b). It can be concluded that if the surface of the membrane is lipophilic, the oil droplets should be spread out on the surface under pressure. However, the current experimental phenomenon can fully show that the as-prepared PDA@PAN nanofibrous membrane has underwater oleophobicity. Lastly, the chloroform droplet was lifted until it was completely separated from the surface of the fiber membrane, and the oil droplet remains intact without mass loss caused by membrane adsorption, as shown in Figure 7c,d. The experimental dynamic photos directly and vividly reflected underwater oil repellency of the as-prepared PDA@PAN nanofiber membrane. It is because the addition of PDA improves the anti-fouling performance of PAN membrane. Hydrophilic groups such as hydroxyl and amino groups in PDA allow the membrane surface to form hydrophilic coatings, avoiding direct contact between oil and membrane.

In order to further verify the superhydrophobic properties of the membrane in water, another experiment was designed. The main difference lies in the continuous release of oil droplets by ordinary needle tubes to simulate the underwater hydrophobicity of membranes under the condition of emulsified oil flow. The experiment was recorded with a digital camera. The video of the process and the staged dynamic pictures collected are shown in Figure 7e–h. It can be observed from the figure that the oil-red dyed n-hexane was quickly injected onto the surface of the nanofibrous membrane in the water medium, and the oil droplets immediately bounced back after contacting the membrane without any adhesion or spreading on the membrane surface. The experimental results once again show that the as-prepared PDA@PAN nanofibrous membrane has good underwater superoleophobicity, combined with its hydrophilic properties, which can endow its suitability for the effective separation of small size oil droplets in emulsion.

### 3.4. Separation Performance of PDA@PAN Nanofibrous Membrane for Oil-In-Water Emulsion

**Figure 8:**
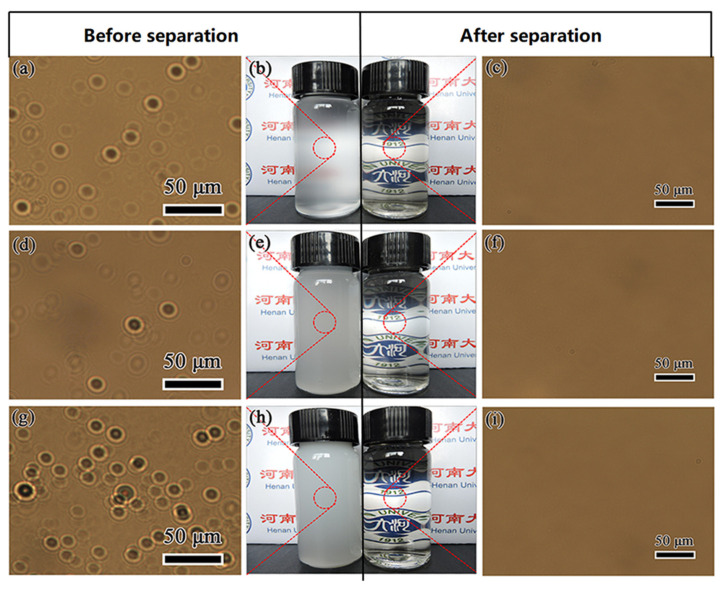
Optical microscopes and digital camera photos of different oil-in-water emulsions: (**a**–**c**) n-hexane/water, (**d**–**f**) n-dodecane/water and (**g**–**i**) n-hexadecane/water before and after separation.

**Figure 9:**
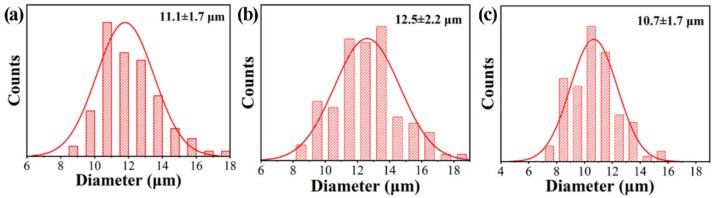
Histograms of size distribution for oil droplets in different oil-in-water emulsions: (**a**) n-hexane/water, (**b**) n-dodecane/water and (**c**) n-hexadecane/water before separation.

Using optical microscopes to observe and analyze the difference of emulsion before and after separation can directly reflect the separation performance of the membrane. Figure 8 shows the optical microscope and digital camera photos of different oil-in-water emulsions such as (a–c) n-hexane/water, (d–f) n-dodecane/water and (g–i) n-hexadecane/water before and after separation. It can be observed from Figure 8a,d,g that before separation, there are many tiny oil droplets in the emulsion that can fill the entire field of view. They are organic droplets coated by the surfactant of SDS and stably dispersed in the continuous aqueous phase. Due to the small diameter of organic droplets (as shown in Figure 9, for the emulsion system of n-hexane, n-dodecane and n-hexadecane, the average diameter of the oil droplets is approximately 11.1, 12.5 and 10.7 μm, respectively), and the dispersion and stabilization of the surfactant, an oil-in-water emulsion was formed, which is opaque in appearance but without oil/water phase separation, as shown in the small reagent bottle on the left in Figure 8b,e,h. It is difficult to separate these emulsions by ordinary oil–water separation materials and methods. However, the as-prepared PDA@PAN nanofibrous membrane in this study can effectively separate them, and the intuitive effect is shown in the right images of Figure 8b,e,h. After the emulsion is completely separated, the filtrate is clear and transparent, indicating that the organic droplets in the emulsion are completely blocked by the filter membrane. As shown in the optical microscope pictures of the filtrate after separation in Figure 8c,f,i, there are no residual organic droplets in the aqueous phase. The experimental results directly show the separation performance of PDA@PAN nanofibrous membrane for oil-in-water emulsion.

### 3.5. Separation Efficiency of PDA@PAN Nanofibrous Membrane for Emulsified Oil

**Figure 10:**
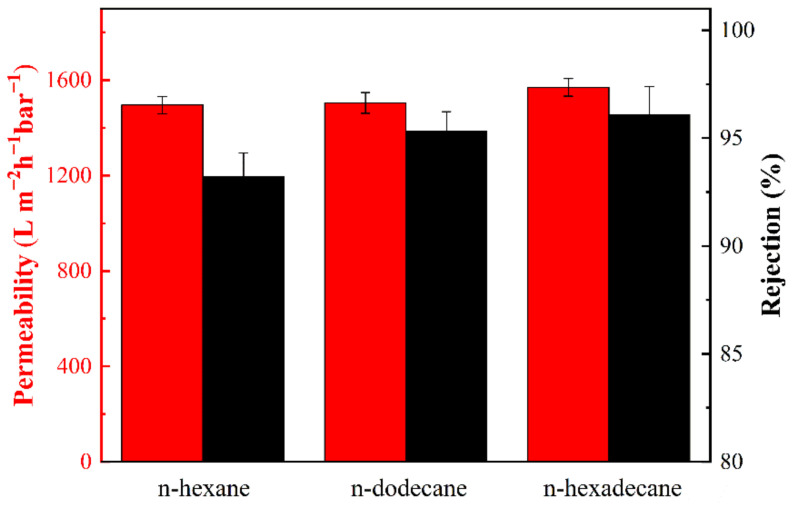
Permeability and separation efficiency of PDA@PAN nanofibrous membrane for oil-in-water emulsions of n-hexane/water, n-dodecane/water and n-cetane/water.

In order to evaluate the specific properties of the prepared PDA@PAN electrospun nanofibrous membrane for oil-in-water emulsion separation, two indexes of water permeability and separation efficiency (rejection) were measured and calculated. Figure 10 shows the measured permeability and separation efficiency of the PDA@PAN nanofibrous membrane for three emulsions of n-hexane/water, n-dodecane/water and n-hexadecane/water. It can be observed from Figure 10 that the permeability of PDA@PAN electrospun nanofibrous membrane for n-hexane/water emulsion is 1496 L m^−2^ h^−1^ bar^−1^, and the separation efficiency is 93.2%. The permeability and separation efficiencies for n-dodecane/water are 1505 L m^−2^ h^−1^ bar^−1^ and 95.3%, respectively, and they are 1570 L m^−2^ h^−1^ bar^−1^ and 96.1% for n-hexadecane/water. All these values are superior to our previous research study [51]. The results show that the permeability and separation efficiencies of the PDA@PAN nanofibrous membrane for n-hexane/water, n-dodecane/water and n-hexadecane/water emulsions have been improved sequentially. There are two main reasons for this: size exclusion and electrostatic interactions. The larger the size of oil-in-water emulsion, the higher the separation efficiency. It is reasonable that as the chain of organic molecules becomes longer, the influence of SDS on their dispersion and stability is weakened, which results in the larger diameter of oil droplet formed by high alkane; thus, it becomes easier to separate. Meanwhile, PDA@PAN nanofibrous membranes are negatively charged, the oil-in-water emulsion dispersed by SDS is also negatively charged and the membrane and oil droplets repel each other during the separation process, thus enhancing oil–water separation performance of the PDA@PAN nanofiber membrane [52,53].

**Figure 11:**
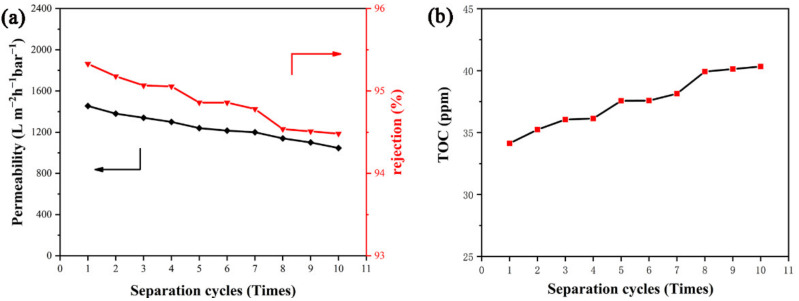
The relationship curves of permeability and rejection (**a**) and TOC (**b**) versus separation cycles of PDA@PAN nanofibrous membrane for n-dodecane/water emulsion.

In the actual application of emulsion separation, recycling reusability is another important requirement for separation materials. Herein, taking the n-dodecane/water emulsion system as an example, the effect of cycle times of PDA@PAN electrospun membrane on its separation performance is investigated. Figure 11 shows the relationship curves of permeability and rejection (a) and TOC (b) versus separation cycles of PDA@PAN nanofibrous membrane for n-dodecane/water emulsion. It can be observed from Figure 11 that as the number of separations increases, the TOC in the filtrate gradually increases (Figure 11b), while permeability and rejection (Figure 11a) decrease. This is because as the number of repeated uses of the separation membrane increases, its separation capacity will inevitably be lost. It may also be due to swelling of the fibers during repeated testing, resulting in additional transport resistance [54]. However, compared with the TOC value (36 ppm) of the filtrate after the first recycling cycle, even after the number of cycles is increased to 10 times, the TOC value of the filtrate is only 40 ppm. Similarly, its permeability only dropped from 1320 to 1199 Lm^−2^ h^−1^ bar^−1^. It is implied that the as-prepared PDA@PAN nanofibrous membrane has good durability for the separation performance of oil-in-water emulsions, which provides a strong guarantee for the practical application of reusable emulsion separation.

## 4. Conclusions

In this paper, firstly, the PAN electrospun nanofibrous membrane was prepared by the electrospinning method, and then PDA nanoparticle coating was fabricated in situ on the surface of PAN fiber by controlling the self-polymerization of DA through a simple and convenient impregnation method. Next, the morphology and structure of the as-prepared PDA@PAN electrospun nanocomposite fibrous membranes were characterized by technologies of SEM, EDX, XPS and FTIR. The unseparated emulsion and the separated filtrate were tested with a total organic carbon analyzer, and the TOC value is used to characterize separation efficiency. Furthermore, the performance of its recycling separation was investigated. The results show that PDA nanospheres are firmly attached to the surface of PAN nanofibers by using the strong chemical binding force between the catechol and amine groups in the mussel-like mucin molecular structure and the substrate. On the one hand, it significantly improved the hydrophilicity of PAN. In addition, it enhances the lasting stability of hydrophilic and oleophobic effects. The optical microscope and actual photos of several oil-in-water emulsions before and after the separation of n-hexane/water, n-dodecane/water and n-hexadecane intuitively reflect that the as-prepared PDA@PAN electrospun nanofibrous membrane has a remarkable separation effect for emulsified oil. The measurement results of the three kinds of emulsion separation efficiency and permeability value clearly show that the filter membrane has excellent emulsion separation performance. Furthermore, by investigating the relationship between the permeability, separation efficiency and TOC value and the number of cycles for the n-dodecane/water emulsion system, it was observed that the super hydrophilic and underwater super oleophobic properties of the membrane exhibited good durability. After 10 times of cyclic separation, the separation ability still has no significant decline. The above results indicate that the as-prepared PDA@PAN electrospun nanofibrous membrane has potential application value in the field of separation for oil-in-water emulsions.

## Data Availability

Data can be available upon request from the authors.
